# Proximity-based proteomics reveals subcellular targeting sequence in Cyanobacteria

**DOI:** 10.1093/plphys/kiaf237

**Published:** 2025-09-08

**Authors:** Maneesh Lingwan

**Affiliations:** Plant Physiology, American Society of Plant Biologists; Donald Danforth Plant Science Center, St. Louis, MO 63132, USA

Cyanobacteria are oxygenic phototrophs that significantly contribute to the carbon and nitrogen cycles by producing O_2_ and fixing N_2_. Unlike most prokaryotes and gram-negative bacteria, cyanobacteria possess a distinctive intracellular organization that includes a plasma membrane (PM), a periplasm and outer membrane with a peptidoglycan layer in between, thylakoid membranes (TMs), and proteinaceous organelles like carboxysomes, which support the carbon-concentrating mechanism (Cameron et al. 2013; Dahlgren et al. 2021). These compartments contain unique proteins essential for various physiological functions, such as photosynthesis, carbon fixation, and nutrient transport; however, the mechanisms for targeting proteins to these locations have remained poorly understood.

Proteomics involves assessing protein interactions, functions, compositions, structures, and their cellular activities. Traditional proteomic analyses of cyanobacterial compartments have relied on biochemical fractionation and separation techniques. These approaches have limitations regarding cross-contamination between fractions, resulting in uncertain cellular localizations. To address these challenges, in this current issue of *Plant Physiology*, Dahlgren et al. (2025) employed proximity-based proteomics techniques using ascorbate peroxidase (APEX2) in the model cyanobacterium *Synechococcus* sp. PCC 7,002 to identify subcellular proteomes (Figure A). APEX2 catalyzes a reaction using biotin-phenol and hydrogen peroxide as substrates, generating biotin-phenol radicals that covalently bind to proteins within a 20-nm radius from the site of production (Figure B). This method allows for specific labeling of membrane-bound proteomes without requiring prior cell lysis, thus saving time and minimizing cross-contamination and limitations (Rhee et al. 2013).

**Figure.**
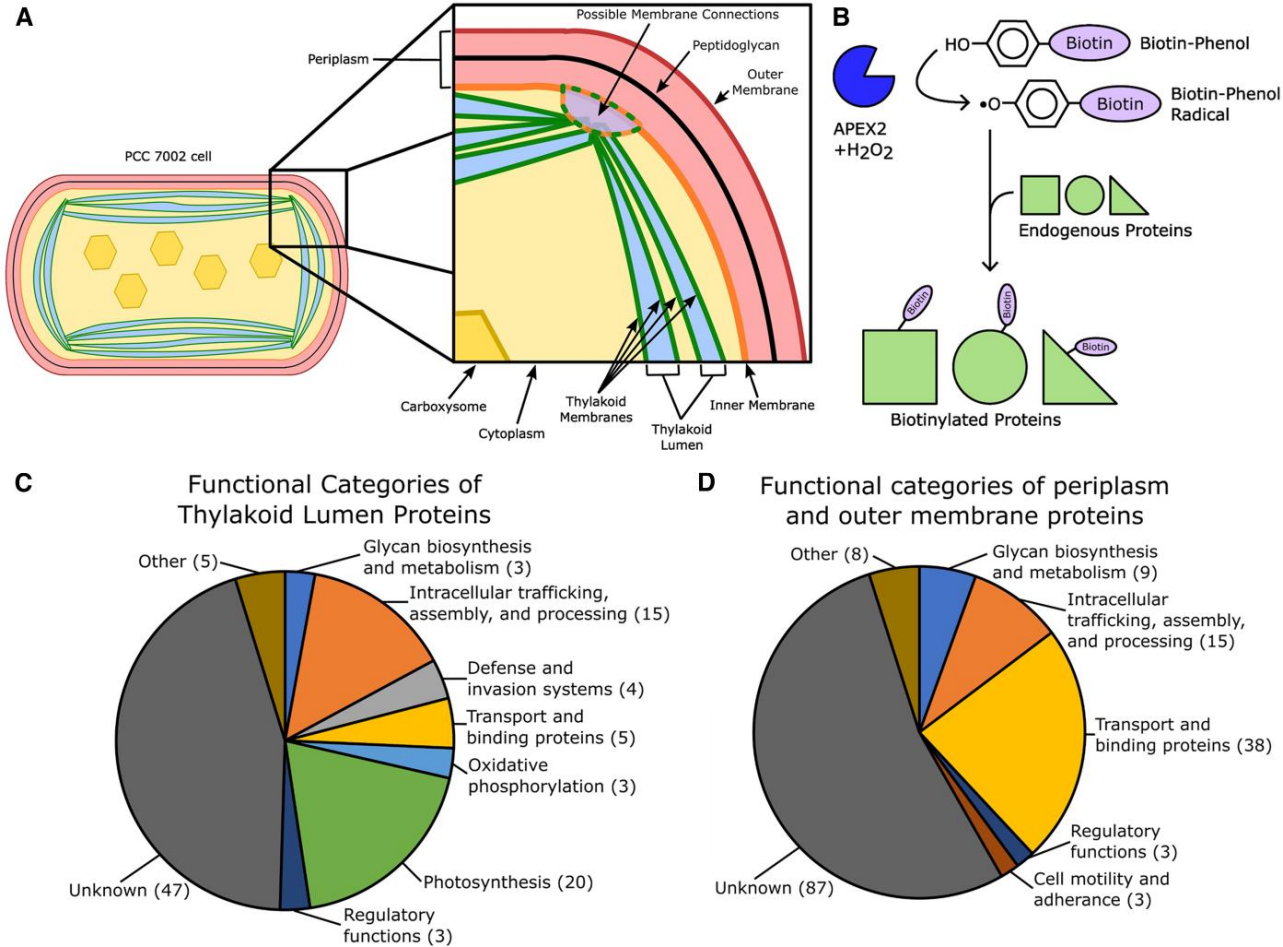
Proximity-based proteomics using APEX2 of cyanobacteria *Synechococcus* sp. PCC 7,002. **A)** Color-coded illustration showing the cellular and membrane structure of PCC 7002. The periplasmic space, which contains the cell wall, is surrounded by inner and outer cell membranes. TMs encase the thylakoid lumen. The zoom box depicts potential transient membrane connections between the inner and TMs. Hexagons within the cytoplasm represent carboxysomes. **B)** In the presence of biotin-phenol (BP) and hydrogen peroxide (H_2_O_2_) substrate, APEX2 catalyzes a reaction creating a BP radical. The BP radical covalently labels endogenous proteins. The functional classifications of proteins localized to **C)** thylakoid lumen, **D)** periplasm, and outer membrane proteome.

Dahlgren et al. (2025) demonstrated that APEX2-based proteins were active in multiple cellular compartments, such as the cytoplasm, thylakoid lumen, and periplasm and outer membrane (P-OM). One important finding was the specific signal sequence assigned to each subcellular compartment. For instance, APEX2 can be fused with extrinsic photosynthetic subunits of PSII such as luminal marker proteins PsbU and PsbQ, and A1097 and A1761 for labeling periplasmic marker proteins. Proteome enrichment analysis confirmed that the thylakoid lumen was enriched in proteins related to photosynthesis and the assembly of photosynthetic complexes, confirming its role in energy generation and oxygen-evolving complex proteins (Figure C). In contrast, P-OM proteome contained factors implicated in metabolite transport, cell wall maintenance, motility, and cell adhesion. Dual localization of 40 proteins was found in both the thylakoid lumen and P-OM proteomes, suggesting shared or transitional roles that might support TM biogenesis (Figure D).

Proximity-based proteomics approaches can be utilized to understand how proteins are directed to their respective compartments. In cyanobacteria, proteins are translocated across membranes either via the secretory (Sec) pathway to translocate unfolded proteins or the twin-arginine translocation (Tat) pathway to translocate folded proteins (Frain et al. 2016). Proteins exported by the periplasm and the thylakoid lumen utilize N-terminal signal sequences that guide their localization. These sequences are cleaved by different signal peptidases (SPI, SPII, or SPIII) that recognize distinct motifs. Most cyanobacteria have 1 set of genes for the Sec and Tat translocation systems, which are localized to the PM or TM (Russo and Zedler 2020), raising questions about how proteins are specifically targeted to the correct compartment. Dahlgren et al. (2025) suggested that signal sequences possess distinguishable characteristics that can recognize translocation complexes in the PM and TM. Signal sequence analysis revealed a notable difference between proteins localized to the thylakoid lumen and those localized to the periplasm. In PCC 7,002, thylakoid lumen-targeted proteins translocated by the Sec pathway typically possessed more hydrophobic and alpha-helical H-regions in their signal sequences compared to P-OM proteins. Interestingly, this trend of signal peptides was conserved among other thylakoid-containing cyanobacteria, such as *Synechocystis sp.* PCC 6,803 and *Nostoc sp.* PCC 7,120, but not in *Gloeobacter violaceus* PCC 7,421, a species that lacks internal TMs. These conserved peptides indicate that the adaptations of the signal sequence coevolved with the development of TM systems.

APEX2 labeling-based proteomic analysis also revealed subdomains within major compartments. Distinct bait proteins present within the same compartment, such as the cytoplasm or periplasm, were associated with overlapping yet distinct proteomes, suggesting a degree of functional sub-organization. The relationship between APEX2 labeling in the periplasmic and cytoplasmic areas was stronger than the correlation between the cytoplasm and thylakoid lumen. This variation is due to the Tat signal sequences in periplasm-targeted bait proteins (A1097, A1761) facilitating post-translational translocation of fully folded proteins into the periplasm. In contrast, Sec pathway substrates like thylakoid-targeted fusions (PsbQ) remain unfolded and minimize cytoplasmic exposure and activity of APEX2. A total of 1,687 proteins were identified, of which 271 were unlocalized. These unlocalized proteins are possibly integral membrane proteins, for which the APEX2 labeling across the membrane poses challenges to precise localization.

While APEX2 is highly suited for soluble proteins within membrane-bound compartments, it is less effective with integral membrane proteins, probably due to the varying accessibility of biotinylation sites. The study also identified a subset of 27 dually localized proteins of unknown function, highlighting potential new players in membrane biogenesis and photosystem assembly. Previous studies have limitations in identifying the hydrophobic H-region trend, likely due to smaller datasets, lack of signal sequence type separation, and absence of thylakoid lumen-specific proteomic data. Dahlgren et al. (2025) utilized a comprehensive dataset and improved methodology to provide a clearer picture of the signals guiding protein trafficking. The advantage of APEX2-based proximity proteomics has opened new avenues for understanding subcellular organization and quantitative profiling of the compartmentalome of cyanobacteria. This approach can provide targets for synthetic biology for engineering photosynthetic organisms with targeted protein expression.

## Data Availability

No new data included in this article.
